# Assessment of perceived support in the context of emergency: Development and validation of the psycho-social support scale

**DOI:** 10.1007/s12144-022-03344-z

**Published:** 2022-06-26

**Authors:** Anna Panzeri, Ornella Bettinardi, Gioia Bottesi, Giorgio Bertolotti, Luca Brambatti, Michela Monfredo, Giuseppe Mignemi, Giovanni Bruno, Giulio Vidotto, Andrea Spoto, Paola Frattola, Silvia Chiesa

**Affiliations:** 1 Department of General Psychology, University of Padua, via Venezia 8, Padua, PD Italy; 2 Department of Mental Health AUSL of Piacenza, Via Anguissola, 15 Piacenza, Italy; 3 Primary Care Psychology, Arona, Novara, Italy; 4 Hospital Guglielmo da Saliceto, Via Taverna 49, Piacenza, Italy; 5 Primary Care Department, AUSL of Piacenza, Via Anguissola 15, Piacenza, Italy

**Keywords:** Assessment, Perceived psycho-social support, Psychological intervention, Emergency, Psychology, Clinical psychology

## Abstract

In research and clinical contexts, it is important to briefly evaluate perceived Psychological and Social Support (PSS) to plan psychological interventions and allocate efforts and resources. However, an appropriate brief assessment tool for PSS was lacking. This study aimed at developing a brief and accurate scale to specifically measure PSS in clinical and emergency contexts, with specific, relevant, targeted, and irredundant items. Experienced clinicians developed the perceived Psycho-Social Support Scale (PSSS) and administered it to a clinical sample (N = 112) seeking psychological help during the COVID-19 emergency. A Confirmatory Factor Analysis examined the PSSS internal structure, and a Multiple Indicator and Multiple Causes model investigated its association with the number of sessions and emotional symptoms. The PSSS showed good psychometric properties and the Confirmatory Factor Analysis provided acceptable fit indexes for a unidimensional structure. The Multiple Indicators and Multiple Causes revealed that more sessions and emotional symptoms were associated with lower PSSS scores. The PSSS is a reliable brief tool to measure PS and could be useful to individualize treatments (i.e., number of sessions) to efficiently allocate efforts and resources in clinical contexts and emergencies (e.g., earthquake, COVID-19 pandemic).

**Supplementary Information:**

The online version contains supplementary material available at 10.1007/s12144-022-03344-z.

## Introduction

Since 2020, the COVID-19 pandemic had an outstanding psychological impact with pervasive psychological discomfort (Bruno et al., 2020; Xiong et al., 2020) up to severe psychological difficulties (e.g., anxiety and depression) whose prevalence significantly increased over the entire population (Cooke et al., 2020).

Therefore, several individuals contacted the hospital’s mental health services seeking psychological help. In this emergency context, psychologists need to provide timely and effective interventions addressing the psychological needs of a large number of people during the health emergency.

Emergency psychology guidelines (WHO, 2020) recommend prompt interventions to relieve and manage discomfort. Effective large-scale psychological interventions in the emergency should properly allocate resources by distinguishing two levels of support depending on the level of individual need (CNOP, 2020).

To do so, vulnerability and protective factors to reduce stress and enhance individual resources should be considered. Among vulnerability and risk factors, some persons experienced traumatic events such as contagion or deaths of loved ones. Additionally, the life-threatening COVID-19 can generate intense negative emotions, such as fear that can intensify anxiety (e.g., of illness, death), which can trigger depressive symptoms (Freeston et al., 2020).

Among the resources and protective factors, the perceived Psycho-Social Support (PSS) is the most relevant. PSS is the subjective perception that psychological and social support are available and effective when needed. According to Wethington and Kessler (1986) and more recent studies (McDowell & Serovich, 2007), perceived PPS may be more important than the actually received support. Indeed, some individuals may display diminished sensibility and responsivity to PSS (due to preexisting conditions such as higher distress and trauma). The literature highlighted that PSS can buffer the adverse effects of stress (Greenberg et al., 2003) also in illness-related contexts (Engel et al., 2021) and that its lack is associated with distress symptoms (Rossi et al., 2022; Zhang et al., 2019). Despite valuable social support from family and friends is a protective factor against stress and traumatic events, during the COVID-19 pandemic, lockdown and social distancing undermined social support, and its lack was significantly associated with distress - highlighting its relevance (Nese et al., 2020; Parola et al., 2022; Ratti et al., 2017; Szkody et al., 2020; Yang & Jiang, 2020).

Thus, PSS is important in predicting adjustment to stressful events, also, it is useful to plan the intervention length (Kataoka-Yahiro et al., 1996). Indeed, according to the Good-Enough-Level (GEL) model, individuals who change faster, will reach a faster satisfactory outcome, and will thus have shorter treatments (Falkenström et al., 2016). Conversely, individuals with lower perceived PSS may need longer and more structured psychological treatments.

Evaluating the ability to perceive PSS is extremely useful for clinicians to plan efficient large-scale emergency interventions by identifying individuals requiring more structured psychological interventions. However, PSS is too often neglected in psychological assessment, most of the studies assessing it by using single-item or ad hoc measures (Engel et al., 2021; Kataoka-Yahiro et al., 1996; McDowell & Serovich, 2007). To date, a brief assessment tool for PSS is lacking.

Of note, one of the most used tools for assessing the positive outcome of an intervention is the so-called “Factor C” or “Positive Change” scale (Anselmi et al., 2015; Michielin et al., 2008) of the *Outcome Questionnaire (‘Valutazione Esito’)* scale of the *Cognitive Behavioral Assessment Battery* (CBA-OE; Bertolotti et al., 2015; Michielin et al., 2008; Sanavio et al., 2013). Factor C includes some items measuring PSS, but it is a relatively long scale as most of its items pertain to constructs other than PSS (e.g., coping strategies, perception of positive change, depression, distress).

Because Factor C does not uniquely assess psycho-social support, it could be the starting point to build an accurate and brief assessment tool. Moreover, among the relevant strengths and advantages of brief measures, they are suitable for contexts with time-constraint limitations (e.g., emergency) and agile to be easily included in longer assessment batteries and very large booklets together with several other measures.

Thus, an accurate and brief psychodiagnostic assessment tool to precisely assess PSS was lacking - and was particularly needed in the first phase of the COVID-19 pandemic.

### The Present Study

Considering this background, this research aimed to develop and preliminarily validate a brief scale to uniquely assess PSS - in a suitable way for emergency contexts. Factor C was the starting point to develop a more tailored measure of PSS, the Psycho-Social Support Scale (PSSS).

First, the psychometric properties and internal structure of the PSSS were tested. Second, we examined the relationships of the PSSS with other constructs associated with PSS.

Regarding the research hypotheses, higher PSS was expected to be associated with:

- *hp#1)* higher personal protective resources (e.g., coping strategies, familiar support, willingness to receive help, accepting reassurance) (Kunzler et al., 2021);
- *hp#2)* lower emotional distress symptoms (e.g., anxiety, sadness);
- *hp#3)* fewer clinical sessions are needed to restore an acceptable psychological condition.
- *hp#4)* we expected a positive association between PSS and the satisfaction with the psychological intervention provided.

## Methods

### Development of the Psycho-Social Support Scale

The item pool for the *Psycho-Social Support Scale* (PSSS) was selected starting from the Positive Change (PC) scale of the CBA-OE (Bertolotti et al., 2015; Michielin et al., 2008). The PC scale is made up of 11 items measuring the perception of positive change, coping with difficulties, and getting others’ psychological support.

A pool of three expert psychologists of the CBA group and the EPE team independently examined the clinical content of the items of the PC scale to select the ones specifically evaluating the PSS. Items related to other constructs - experienced difficulties, coping abilities - were discarded. There were no disagreements among judges (Cohen’s Kappa for inter-rater agreement = 1). Table 1 shows the items of the original PC scale and the newly developed PSSS.

**Table 1:** Items of the original scale and those of the new psycho-social support scale in bold

**#**	Item	Included in the PSSS	
1	**I felt helped by others**	✔	
2	**I felt understood by others**	✔	
3	I felt overwhelmed by difficulties		✘
4	I felt able to react even to difficulties and failures		✘
5	I got the feeling that the worst was over		✘
6	I trusted myself		✘
7	I have seen possibilities for solutions to my problems		✘
8	**I was able to talk to others**	✔	
9	I tried to face the difficulties rather than avoid them		✘
10	**Someone helped me solve my personal problems**	✔	
11	I am satisfied with the goals I have achieved or am about to achieve		✘

Items included in the new version are highlighted in bold

Table 2 reports the Italian version of the PSSS with the instructions for administration and scoring.

**Table 2:** Italian version of the psycho-social support scale

Istruzioni: legga le seguenti frasi e per ognuna segni la risposta che meglio descrive come si è sentito in questo periodo. Faccia riferimento agli ultimi 15 giorni, compreso oggi, e scelga la sua risposta tra queste.						
#	Item	Per nulla	Poco	Abbastanza	Molto	Moltissimo
1	Mi sono sentito aiutato dagli altri	0	1	2	3	4
2	Mi sono sentito capito dagli altri	0	1	2	3	4
3	Sono riuscito a parlare con gli altri	0	1	2	3	4
4	Qualcuno mi ha aiutato a risolvere i miei problemi personali	0	1	2	3	4

Per calcolare il totale, sommare i punteggi degli item. Il punteggio massimo è 16

Respondents are asked to ‘Read each of the following sentences and mark the answer that best describes how you have felt over the past 15 days, including today.’ In line with the original PC, the PSSS items were rated on a 5-point Likert-type scale ranging from 0 to 4 (0 = ‘not at all’, 1 = ‘a little bit’, 2 = ‘somewhat’, 3 = ‘a lot’, 4 = ‘very much’). The total score can be easily computed by summing the items, it ranges between 0 and 16, with higher scores indicating higher PSS.

Then, the newly developed PSSS was administered to participants within a psychological intervention with the following procedure.

### Participants

Participants were consecutively enrolled from the general population living in Emilia Roma gna – a “red” area since February 2020 when COVID-19 violently spread in Italy.

The inclusion criteria were: (I) being a native Italian speaker; (II) seeking psychological help to the the EPE at the Department of Mental Health of the Hospital of Piacenza since the first phase of the COVID-19 emergency; (III) taking part in at least one psychological session. The exclusion criteria were: (IV) lack of consent to use data for research aims and (V) age < 18 years.

### Procedure

Participants were enrolled from people who received psychological intervention during the COVID-19 pandemic. Indeed, the hospital reactivated the pre-existing Equipe for Psychological Emergency (EPE) – n. 30 experienced psychologists and psychotherapists - that conducted the below-described psychological intervention that used the PSSS.

### Psychological Intervention

The intervention was created to be delivered both at distance (e.g., by telephone) and in face-to-face settings. The intervention encompassed the psychological assessment of distress and resources. In a CBT-oriented framework, the intervention used emergency psychology practices (Solomon & Hensley, 2020).

This brief initial intervention (up to 4 psychological sessions) aimed to provide immediate relief from stress and negative feelings, promote bereavement elaboration, and in the ‘*indication for action’* part identify the needed treatment level (first or second). According to the patient’s condition, the clinician conducted a number (from 1 to 4) of individual psychological sessions to restore an acceptable psychological help - patients needing more sessions had more difficulties.

Then, the clinician indicated the treatment level: first level treatment was indicated if the patient did not need further support after the fourth session; second level treatment was indicated if the patient needed further support after the fourth session, resulting in being redirected to the territorial services or psychiatry units.

#### Measures

##### The Psycho-Social Support Scale (PSSS)

It is a self-report questionnaire to measure the perceived level of PSS. Respondents are asked to mark the answer that best describes how they felt over the past 15 days. The PSSS has 4 items rated on a 5-point Likert-type scale ranging from 0 to 4 (0 = ‘not at all’, 1 = ‘a little bit’, 2 = ‘somewhat’, 3 = ‘a lot’, 4 = ‘very much’). The total score is the sum of the items (range 0–16). Higher scores indicate higher levels of PSS.

##### The Level of Satisfaction with the Aid Received

It was measured with a single question from the CBA-OE Factor C, namely *“How helpful do you think our psychological support has been to you?”* with a Likert-type format from 0 (= not at all) to 4 (= very much).

##### The Checklist

An ad-hoc semi-structured checklist created by the EPE helped clinicians in the psychological intervention. The checklist (see Appendix for the English and Italian version) has a total of 57 items divided into 3 parts.

Part #1, administered only in the first session with the EPE team, includes:

- socio-demographic characteristics;
- psychiatric history (9 items);
- two open questions about the subjective experience leading to seeking psychological help (i.e., *“Can you tell me what happened to you/your experience?”*, *“What were the reactions experienced at the beginning and during the event (contagion/recovery, news of death)?”*); and personal resources (i.e., *“What or who helped/is helping you cope with the event?”, “In the hours and days that followed, what brought you some relief and help?”, “Each of us has developed and honed personal strategies over time to reduce stress at critical times in our lives. What strategies have been helpful to you in the past during difficult times?”*).

Part #2 can be used in each session and evaluates:

- Psychological distress, including:
  *PTSD symptoms*: as the sum of 3 dichotomous (1 = present, 0 = absent) items about avoidance, intrusivity, and hyperarousal.
  *cognitive symptoms: as* the sum of 5 dichotomous items (1 = present, 0 = absent) about problems in memory, concentration, problem-solving, denial defense, sense of unreality, or muffling;
  *behavioral symptoms:* as the sum of 6 dichotomous items (1 = present, 0 = absent) about self-closure/isolation, avoidance, aggression, changes in eating habits, self-medication with substances, sleep difficulties;
  *emotional symptoms:* as the sum of 7 dichotomous items (1 = present, 0 = absent) about helplessness, anger, sadness, anxiety, depression, emotional numbness, and irritability;
  *The severity of depression and anxiety:* as the sum of 2 items scored on a three-point scale (absent = 0, mild = 1, severe = 2) continuum according to the level of impairment caused to the person,
- Positive resources:
  *resources or protective factors*: as the sum of 4 dichotomous items (present =1, absent = 0) about coping strategies, absence of socioeconomic stress, willingness to accept psychological help, accepting reassurance;
  *extended social support*: as the sum of 4 dichotomous items (present =1, absent = 0) about family, friends, religious community, and health professionals.
- the *type of psychological intervention*: with 9 dichotomous categorical items (1 = used, 0 = not used) about stabilization, normalization, psychoeducation, adaptive coping skills, counseling, bereavement support, referral to another service, COVID-related information, and network intervention.
- Indications for action and priority of intervention:

- in the *indication for the action* part, the clinician identified the needed treatment level between:
  I. *first level -* continue with the EPE for the acute phase (maximum 4 sessions);
  II. *second-level –*medical intervention, referral to the Department of Mental Health and Pathological Addictions (e.g., psychiatry), or other in the short-long term.
- the *priority of intervention* was rated on a three-point scale with:

- green code: mild symptoms not requiring urgent intervention;
- yellow code: moderate symptoms requiring monitoring;
- red code: severe symptoms requiring immediate specialized intervention.

Part #3, administered only in the last session with the EPE team, including the above-described tools:

- the newly developed *PSSS scale*,
- the *level of satisfaction with the aid received*.

### Ethics Statement

The research procedure was in line with routine practices applied by the hospital in an emergency, was approved by the Scientific Direction of the Hospital of Piacenza, Italy, and was conducted according to the Helsinki guidelines. All participants were informed about the study aims, voluntarily agreed to participate, and provided informed consent to use their anonymized data for research aims.

### Statistical Analysis

The *R* software was used with the packages ‘*psych’* (Revelle, 2015), ‘*lavaan’* (Rosseel, 2012), and ‘*semPlot’* (Epskamp, 2022)*.* Descriptive statistics displayed the sample demographics and psychological characteristics. Then, the validity of the PSSS scale was studied.

First, the item psychometric properties were examined – skewness, kurtosis, and item-total correlation. Cronbach’s α assessed the scale internal consistency (desirable value > .70).

Second, a confirmatory factor analysis (CFA) was conducted to confirm the factor structure of the PSSS.

Third, the Multiple Indicators and Multiple Causes (MIMIC) model – a mixed-modeling technique – investigated the influence of two observed exogenous continuous predictor variables (*number of clinical sessions* with the psychologist and number of emotional symptoms) on the latent variable (PSS) (Kline, 2015). Modification indices (MI) were considered. Finally, the path coefficient between the predictor and the latent factor variable evaluated the impact of the number of sessions on PSS.

For the CFA and the MIMIC, the same estimator and fit indexes were used. Given the response scale, the diagonally weighted least squares (DWLS) estimator was used for its suitability to both Likert and dichotomous scales (Consoli et al., 2020; Manzoni et al., 2021; Milavic et al., 2019; Pietrabissa et al., 2020; Rossi Ferrario et al., 2019).

The following cutoffs indicating ‘acceptable’ model fit were applied: (a) the Satorra-Bentler χ2 (S- Bχ2) and (b) the chi-square statistic (χ2) should be non-statistically significant (*p* > .05); (c) the χ2 divided by the degrees of freedom (χ2/df) should have values of 3 or less; (d) the Tucker-Lewis fit index (TLI) and (d) the comparative fit index (CFI) – both TLI and CFI values approximating at least 0.95 indicate good fit; (e) the root mean square error of approximation (RMSEA) with values <0.06 were supposed to demonstrate acceptable model-data fit, and (f) the RMSEA 90% confidence interval (CI) containing 0.05 indicated the possibility of close fit (Browne & Cudeck, 1993); (e) the Standard Root Mean square Residual (SRMR) should be <0.080 (Muthén and Muthén, 1998–2017; van de Schoot et al., 2012; Brown, 2015; Kline, 2015; Hu & Bentler, 1999).

Fourth, regressions (a) investigated the convergent validity of the PSSS on the satisfaction with the received psychological help and (b) explored the predictors (emotional symptoms, level of anxiety, personal resources) – of the PSS (outcome).

## Results

Table 3 describes the characteristics of the sample of 112 individuals seeking psychological help (76% females; mean age = 57.1, SD = 13.29). Most (82.1%) were caregivers of the deceased because of COVID-19, the others had COVID-19 disease isolated at home or in hospital. Most of the sample (94.6%, N = 106) contacted the EPE team by telephone and video call.

**Table 3:** Descriptive statistics of the sample (*n* = 112)

Variable	N (%)
Sex	
Males	27 (24.1%)
Females	85 (75.9%)
Type	
C19 patient	20 (17.9%)
C19 Caregiver	92 (82.1%)
Contact	
Self	19 (17%)
Contacted by EPE	91 (81.3%)
Other	2 (1.8%)
Month	
March	28 (25%)
April	68 (60.7%)
May	12 (10.7%)
June	4 (3.6%)
Setting	
At distance	106 (94.6%)
In presence	6 (5.4%)
Previous mental health treatments	
Yes	22 (29.6%)
No	90 (80.4%)

Table 4 shows the psychological characteristics of the sample. Despite most people having good personal and social resources, the most common psychological and emotional difficulties were depression, sadness, anxiety, hyperarousal, and denial.

**Table 4:** Psychological characteristics of the sample (*n* = 112)

Variables	*n* (%)
PTSD symptoms	
Avoidance	4 (3.6%)
Intrusivity	11 (9.8%)
Iperarousal	12 (10.7%)
Emotional difficulties	
Helplessness	48 (42.9%)
Anger	22 (19.6%)
Anxiety	48 (42.9%)
Depression	38 (33.9%)
Emotional numbness	11 (9.8%)
Sadness	83 (74.1%)
Irritability	13 (11.6%)
Severity of symptoms	
Mild anxiety	52 (46.4%)
Severe anxiety	60 (54.6%)
Mild depression	48 (42.9%)
Severe depression	64 (57.1%)
Resources	
Coping	87 (77.7%)
Family support	68 (60.7%)
Availability for help	74 (66.1%)
Cognitive difficulties	
Memory difficulties	11 (9.8%)
Focusing difficulties	40 (35.7%)
Problem-solving difficulties	16 (14.3%)
Denial	13 (11.6%)
Sense of unreality	45 (40.2%)
Behavioral difficulties	
Self-closure, isolation	21 (18.8%)
Avoidance	12 (10.7%)
Aggressivity	3 (2.7%)
Changes in eating behaviors	14 (12.5%)
Substance use	7 (6.3%)
Sleep difficulties	39 (34.8%)
Priority of intervention	
Low	70 (62.5%)
Mild	39 (34.8%)
High	3 (2.7%)
Support	
Family	95 (84.8%)
Friends	42 (37.5%)
Religion	11 (9.8%)
Health professionals	16 (14.3%)
Satisfaction with the EPE aid	2.82 (.68)

Table 5 shows that most of the psychological interventions required a first level (lower intensity) with one single clinical interview (66,1%, n 74), whilst 33.9% of patients required a second-level intervention (higher intensity) with more than one clinical interview (max 5, mean 2,87 ± 0.97), interestingly 69% of them were caregivers of patients with COVID-19 who were ill or already deceased. 38.6% of the sample reported mild to high severity of psychological and mental health difficulties requiring a more structured psychological intervention. The most used psychological intervention was psychological support in its several facets, always with empathetic and warm management of emotions and cognitions. Psychological support was focused on bereavement, from its communication up to favoring grief elaboration.

**Table 5:** Psychological interventions: levels and types

	First level	Second level	Total
Users			
Patients	8	12	20
Caregivers	66	26	92
Type of intervention			
Support	12	9	21
Normalization	8	6	14
Psychoeducation	10	6	16
Stress reduction coping strategies	10	2	12
Bereavement communication and support	30	11	41

First level: *n* = 74; second level: *n* = 38

### Psychometric Properties and Validation of the PSSS

Table 6 shows the item properties of the PSSS, all the items showed skewness and kurtosis values within the normal range (between −1 and + 1) and thus are considered acceptable to prove normal univariate distribution. Cronbach’s alpha for the total scale was .776, indicating good internal reliability.

**Table 6:** Psychometric properties of the items of the psycho-social support scale

Descriptive statistics						
	Mean	SD	Skewness	Kurtosis	ITC	α without
PSSS total score	9.20	2.59	−0.274	0.122	–	–
Item #1	2.36	0.826	−0.460	0.567	.717	.694
Item #2	2.20	0.804	−0.479	−0.413	.679	.715
Item #3	2.28	0.750	0.017	−0.232	.497	.799
Item #4	2.37	0.900	−0.193	0.341	.567	.774

*ITC* Item total correlation

#### CFA

A CFA verified how well the data fit the single-factor model with the DWLS estimator. The statistical non-significance of χ^2^ (χ^2^ = 2.104, df = 2, *p* = .349) shows an acceptable fit of the data to the model as well as the χ^2^/df = 2.48. The fit indices considered CFI = 1, TLI = 1, RMSEA = 0.022 (95%CI: 0.000–0.191; *p* = .445), SRMR = 0.038 are acceptable. Table 7 shows the item factor loadings.

**Table 7:** Item factor loadings (λ) and explained variance (R^2^) of the CFA and MIMIC models

CFA model				MIMIC model			
	λ	R^2^	p value		λ	R^2^	p value
Item #1	0.939	0.881	<.001	Item #1	0.921	0.871	<.001
Item #2	0.856	0.733	<.001	Item #2	0.834	0.736	<.001
Item #3	0.609	0.371	<.001	Item #3	0.571	0.370	<.001
Item #4	0.668	0.447	<.001	Item #4	0.672	0.500	<.001
				#Int.	−0.223		<.001
				#Emo.	−0.234		<.001

Note: *CFA* Confirmatory Factor Analysis, *MIMIC* Multiple Indicators and Multiple Causes Model, #Int. = number of clinical sessions; #Emo. = number of emotional symptoms

### MIMIC

**Fig. 1:**
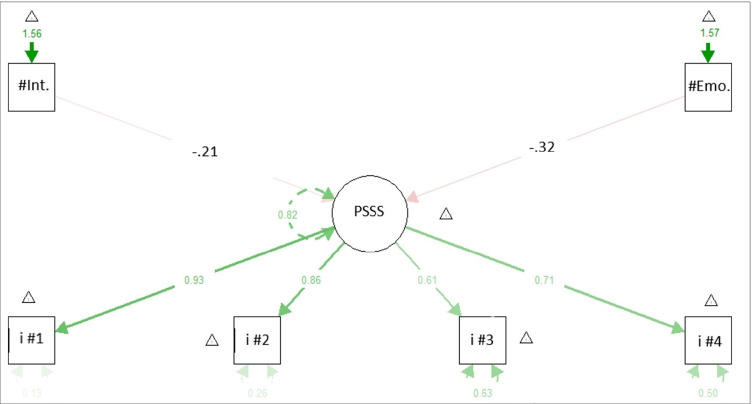
Plot of the MIMIC model with the number of clinical sessions and the number of emotional symptoms as exogenous variables on the PSSS. Note*:* #Int. = number of clinical sessions; #Emo. = Number of emotional symptoms

Further insights into the structure of the model were tested. A MIMIC model was fitted to test the heterogeneity of the population in the latent factor (i.e., PSSS) in relation to a different *number of psychological sessions* and a different *number of emotional symptoms*. The MIMIC, with path coefficients between the covariates and the factor, examined the impact of the number of sessions and the number of emotional symptoms on the latent variable of PSS. The MIMIC showed acceptable model-data fit indexes according to the suggested cut-offs: *χ* ^*2*^ = 6.525, df = 8*, p* = .589; χ^*2*^*/df* = 1.581, CFI = 1, TLI = 1.002, *RMSEA* = 0.000 (90%CI 0.000–0.097, *p* = .753, *SRMR* = 0.043. The covariate had an estimated beta on the latent factor of −0.223 for the *number of sessions* (standard error = 0.067, *p* = .001) and of - .234 for the *number of emotional symptoms* (std. err. .055, *p* = .001). Table 5 shows the factor loadings. Figure 1 shows the MIMIC model structure. The path coefficients in Fig. 1 represent the effect of a covariate on the PSSS, holding constant the other covariates (Kline, 2015). Covariates accounted for about 17.7% of the variance in PSSS scores.

### Convergent Validity of the PSSS

The convergent validity of the PSSS was tested with the Pearson’s correlation coefficient calculated between the PSSS and the perceived satisfaction with the psychological intervention. The correlation was positive and statistically significant (r = .216, *p* = .02).

### Factors Predicting Psycho-Social Support

The linear regression model investigating which psychological factors were associated with perceived PSS explained 23.4% of the variance (R^2^*adj* = .234, F (4.109) = 9.63, *p* < .001). The results showed that both a higher *number of emotional symptoms* (β = −0.16, t (109) = −3.86, *p* < .001) and higher *anxiety* levels (β = −0.58, t (109) = −2.78, *p* < .001) were associated with lower PSSS scores. Furthermore, a higher *number of personal resources* was significantly associated with higher PSSS scores (β = 0.33, t (109) = 4.38,* p* < .001).

## Discussion

The main aim of this study was to develop a new brief scale to uniquely assess PSS in a suitable way for time-constrained contexts (e.g., emergency, hospital, easily fatigued subjects).

The results showed that the PSSS is a reliable and efficient brief scale with good psychometric properties. The internal reliability of the PSSS was very good (Cronbach α = .80) - calculated only with 4 items. The PSSS demonstrated a unidimensional factorial structure supported by acceptable fit indexes in the CFA. Interestingly, the MIMIC model revealed that the *number of psychological sessions* and the *number of emotional symptoms* both had a negative association with the PSS. As in line with research hypotheses, lower levels of PSS are related with experiencing more negative emotions and needing more clinical sessions to feel better. This result is in line with clinical practice in which more efforts (more sessions) are dedicated to the users with higher psychological difficulties and the most critical emotional situations.

Moreover, the PSSS showed interesting associations with some important psychosocial factors and clinical indicators. As expected, the PSS measured with the PSSS was positively associated with higher *personal and social resources*, confirming their protective role. Conversely, higher *emotional difficulties* and higher *anxiety levels* were associated with lower PSS – indeed, during the pandemic, isolated and alone people are more prone to experience lower PSS, captured by the PSSS (Usher et al., 2020). Feeling low perceived support by others (e.g., PSSS) – regardless of the actually received support (McDowell & Serovich, 2007) – may discourage one to seek further support, thus intensifying the emotional symptoms.

Furthermore, the convergent validity of PSSS was good, as expected it showed a statistically significant and positive association with perceived satisfaction with the psychological intervention. This result supports the fourth research hypothesis and it is in line with previous literature reporting that *‘patients most in need of support are the ones less satisfied with the support received’* (Turner et al., 1993). Again, according to the evidence-based literature and the Good-Enough-Level (GEL) model, patients who change faster, will reach a faster satisfactory outcome, and will thus have shorter treatments (Falkenström et al., 2016).

### Implications

The above findings have interesting implications for research practice because the PSS is the first Italian brief tool to measure the perceived PSS, it can be easily integrated into longer assessment batteries, thus reducing the burden and fatigue for participants, allowing to save time – or assess more constructs in the same time.

Regarding the implications for clinical practice, within a broader assessment – the PSS may be useful for clinicians to plan further clinical interventions by effectively allocating resources - precious operators and time - in emergency contexts. Indeed, lower levels of PSS indicate that a person could need more time and effort from health professionals to reach an acceptable level of psychological health, thus individuals with lower PSSS may be those to dedicate more time and resources. Moreover, the described assessment procedure could be used also in other contexts with constrained time-resource ratios (e.g., emergency, public services), and having previously measured the PSS contributes to efficiently planning the clinical path because patients needing further psychological support can be transferred to other services (e.g., psychiatry units).

Moreover, given that the perceived support is usually overestimated rather than underestimated (McDowell & Serovich, 2007), a low level of PSS should be carefully considered. Differently, higher levels of PSS indicate that a person would need less time to feel better, thus allowing clinicians in scheduling better their time and dedicate it to other people in need. This information is extremely useful to allocate and divide resources in emergency contexts with several practical constraints due to the higher patients’ affluence and the limited number of psychologists.

Concerning the intervention phase, the associations of the PSSS with both protective and vulnerability factors were in the expected direction, once more highlighting the importance of promoting the resources and buffering the vulnerabilities influencing PSS. For instance, improving social relationships has positive consequences for individuals, becoming more open and sensible to others’ presence and help. Again, reducing clinical symptomatology (e.g., PTSD, anger) improves the overall disposition to positive changes, thus developing resilient outcomes (Panzeri et al., 2021a). Indeed, scientific literature on psychosocial determinants of health (Kivimäki et al., 2020) shows the mutual interaction between social context factors (health crisis, socioeconomic crisis) and individual and collective psychological factors (stress levels, adaptive strategies, behaviors, etc.). Importantly, this interaction can have repercussions on both psycho-physical health, by leading to elevations of distress levels, impairing psychological well-being, and compromising the social and occupational functioning levels. Moreover, the PSSS may be a suitable tool to be integrated into the preliminary assessment of psychological interventions aimed at favoring the adaptation to a stressful condition (e.g., illness), as well as the cognitive reframing of negative views, and acceptation of the situation (Cattivelli et al., 2018; Giuntoli et al., 2019; Rossi et al., 2021).

### Limitations

This study is not free of limitations that should be considered to disclose fruitful hints for future research. The sample size (*n* = 112), despite sufficient to estimate the statistical parameters, could be enlarged to improve the parameters’ stability. Also, the prevalence of females (76%) did not allow performing measurement invariance and the sample mainly consisted of caregivers actively seeking psychological help. Future studies may try to overcome these points related to the sample characteristics and will extend the validity and generalizability of these results by applying the PSSS also to other populations and in other illness-related contexts. Given the exceptional situation, it was not possible to administer a full battery of assessment tools, so a brief and irredundant checklist was used to save time. Finally, a cross-sectional research design was used because of the fast and time-limited emergency intervention, future longitudinal studies should monitor the evolution of PSS over time in response to a more structured psychological therapy. Despite the soundness of the methodology, the cross-sectional study design does not allow to detect causal relationships among constructs that would require an experimental and/or longitudinal research design.

### Future Research

Future studies may employ the PSSS in other emergency contexts, with different populations, such as the general population not seeking psychological help, young adults, healthcare workers, and at-risk frail patients, who have also shown to suffer the impact of a large-scale emergency such as the COVID-19 pandemic (Balestroni et al., 2020; Panzeri et al., 2021b, c; Parola, 2020; Rossi & Mannarini, 2019; Rossi Ferrario & Panzeri, 2020).

### Strengths

Among the strengths of this study, this research provided the PSSS, a brief and effective tool – that literature was lacking - to uniquely measure the PSS without administering not relevant items, thus it is an appropriate and useful tool for the particular emergency context. Regarding the methodological strengths, this study relied on strong methodology.

The CFA is a well-rooted and precise method to validate measurement tools, further, the MIMIC model revealed that exogenous factors (number of clinical sessions, number of emotional symptoms) were associated with the PSSS scores, disclosing the interest above-discussed implications for clinical settings (e.g., planning interventions). Indeed, those patients with lower levels of PSS and higher emotional symptoms required more sessions.

Moreover, this study described the large psychological distress experienced in the COVID-19 emergency and the need for an effective psychological assessment and intervention that can be used also in other circumstances.

Interestingly, these findings are especially relevant for informal caregivers, since most of the sample were caregivers of patients with COVID-19. Caregivers are too often neglected despite the burden they have to carry (Panzeri et al., 2019; Rossi Ferrario & Panzeri, 2020; Sambasivam et al., 2019). Additionally, during the exceptional first phase of COVID-19 emergency, caregivers suffered because of forced separation from patients, communication difficulties, and grief elaboration (e.g., high rate of death, funeral ban) (Panzeri & Rossi Ferrario, 2020; Rossi Ferrario et al., 2021; Stroebe & Schut, 2021).

## Conclusions

In conclusion, overall this study shows that the PSSS is a brief scale to measure the PSS and can be easily integrated into longer assessment batteries, thus lowering the risk of administering redundant questions to the subject. The PSSS can be useful to plan further psychological treatments and effectively allocate resources in time-constrained settings. Moreover, this research highlights the importance to adapt psychological assessment and measurement tools to specific contexts.

## Supplementary Information

ESM 1 (DOCX 49 kb)

## Data Availability

Restrictions apply to the availability of these data to ensure the privacy of the participants. The data can be requested to the first Author.

## Declarations

All procedures performed in studies involving human participants were following the ethical standards of the institutional research committee of the the Hospital of Piacenza, Italy, and with the 1964 Helsinki Declaration and its later amendments or comparable ethical standards.

### Permission to Reproduce Material from Other Sources

There are no materials reproduced from other sources.

### Clinical Trial Registration

This is not a clinical trial, all procedures were in line with the routine of the hospital.
