# Supplementary material for: Assessment of perceived support in the context of emergency: Development and validation of the psycho-social support scale
Source: Curr Psychol. 2022 Jun 26:1–12. Online ahead of print. doi: 10.1007/s12144-022-03344-z (PMC9243771; doi:10.1007/s12144-022-03344-z)
Supplement: Supplementary file 1 — (DOCX 49 kb) [file 12144_2022_3344_MOESM1_ESM.docx]

**APPENDIX**

# of the article titled:

# Assessment of Perceived Support in the Context of Emergency: Development and Validation of the Psycho-Social Support Scale

**LIST of CONTENTS**

**ASSESSMENT CHECKLIST – English version***……………………………………….…. page 2

**PART 1: first session with EPE team**

GENERAL INFORMATION

**PART 2: for each session with EPE team**

2.A) PSYCHOLOGICAL DISTRESS

2.B) POSITIVE RESOURCES

2.C) INTERVENTION TECHNIQUES

2.D) INDICATIONS FOR ACTION AND PRIORITY OF INTERVENTION

**PART 3: for the last session with EPE team**

**PSYCHO-SOCIAL SUPPORT SCALE – ENGLISH VERSION** …………………. page 6

**PSYCHO-SOCIAL SUPPORT SCALE - VERSIONE ITALIANA** …………….. page 7

-------------------------------------------------------------------------------------------------------------------------------

* The Italian version of the checklist is available under request to the first Author
e-mail: [anna.panzeri@phd.unipd.it](mailto:anna.panzeri@phd.unipd.it)

**ASSESSMENT CHECKLIST – English version***

**Psychologist Name: ...................................... Location: ........... User Code: ...........**

**PART 1: first session with EPE team**

**GENERAL INFORMATION**

**User Contact Date ….…/….…/……… Contact Number…………**

**Surname and first name (of contacted user) …….………………………….……………**

**Gender (user): □ M □ F □ __**

**Age (contacted user) …………**

**User's residence……………………………… TELEPHONE……………………**

**V9) Contact is made by: □ The person (1) □ EPE Psychologist (2) □ Other (3) ...............**

**V10) Past or current treatments**

History of previous psychological interventions □ Yes = 1; □ No = 0

History of previous psychiatric interventions □ Yes = 1; □ No = 0

Current psychopharmacological therapy □ Yes = 1; □ No = 0

**V6) Mode of Contact**

□ Direct in hospital □ E-mail

□ Direct in territorial service □ Telephone

□ Video call □ Other form of contact

**V7) To whom the consultation is provided**

□ General citizen □ Caregiver of deceased

□ Quarantined citizen □ Health professional .....................

□ Hospitalized citizen □ GP/Pediatrician

□ Family member of quarantined person □ Other professional (e.g. social worker)

□ Family member of inpatient □ Other caregiver of patient ....................

**V11) AVAILABILITY for user confrontation: □ (1=YES) available; □ (2=NO) not available**

**PART 2: for each session with EPE team**

**2.A) PSYCHOLOGICAL DISTRESS**

**V10) Narration:** **"Can you tell me what happened to you/your experience? .........................................**

**V13)** **What were the reactions experienced at the beginning and during the event
 (contagion/recovery, news of death)? ………………………………………………………………………………………………**

**Number of PTSD, cognitive, behavioral, and emotional symptoms**

|  |  | **Session number** | | | |
| --- | --- | --- | --- | --- | --- |
|  |  | **1** | **2** | **3** | **4** |
| **PTSD symptoms:**  v12) avoidance |  | **□** | **□** | **□** | **□** |
| v13) intrusivity |  | **□** | **□** | **□** | **□** |
| v14) hyperarousal |  | **□** | **□** | **□** | **□** |
| **Cognitive symptoms:**  v21) memory problems |  | **□** | **□** | **□** | **□** |
| v22) concentration |  | **□** | **□** | **□** | **□** |
| v23) difficulty in solving problems |  | **□** | **□** | **□** | **□** |
| v24) denial - defense |  | **□** | **□** | **□** | **□** |
| v25) sense of unreality or muffling |  | **□** | **□** | **□** | **□** |
| **Behavioral symptoms:**  v33) self-closure/isolation | **D** | **□** | **□** | **□** | **□** |
| v34) avoidance |  | **□** | **□** | **□** | **□** |
| v35) aggression | **A** | **□** | **□** | **□** | **□** |
| v36) changes in eating habits |  | **□** | **□** | **□** | **□** |
| v37) self-medication with substances  (***avoidance***) |  | **□** | **□** | **□** | **□** |
| v38) sleep difficulties |  | **□** | **□** | **□** | **□** |
| **Emotional symptoms**:  v26) helplessness | **D** | **□** | **□** | **□** | **□** |
| v27) anger | **A** | **□** | **□** | **□** | **□** |
| v28) sadness | **D** | **□** | **□** | **□** | **□** |
| v29) anxiety |  | **□** | **□** | **□** | **□** |
| v30) depression | **D** | **□** | **□** | **□** | **□** |
| v31) emotional numbing |  | **□** | **□** | **□** | **□** |
| v32) irritability | **A** | **□** | **□** | **□** | **□** |

**DEPRESSIVE SYMPTOMS** area = true response item 26,28,30,33 (range 0-4) **_____**

**ANGER SYMPTOMS** area = true response item 27, 32, 35 (range 0-3) **_____**

| **SEVERITY OF SYMPTOMATOLOGY *:** | |
| --- | --- |
| **v15) ANXIOUS** | ABSENT (0) MILD (1) SEVERE (2) |
| **v16) DEPRESSIVE** | ABSENT (0) MILD (1) SEVERE (2) |

***** According to the impairment caused to global personal functioning

**2.B) POSITIVE RESOURCES**

**V14) Active resources: "What or who helped/is helping you cope with the event? ………………………**

**V16) “In the hours and days that followed, what brought you some relief and help?" ……………………**

**V16a) Implementation of resources:**

"Each of us has developed and honed personal strategies over time to reduce stress at critical times in our lives." What strategies have been helpful to you in the past during difficult times?.........................................

………………………………………………………………………………………………………………………………………………………….

**V18) Closure: Validation and questions**

“Would you like to add anything or have anything to ask me?” .................................................................

| **EVALUATION OF RESOURCES OR PROTECTIVE FACTORS ____ / 4** | |
| --- | --- |
| **v17) COPING STRATEGIES** □ 0= NO, □1 = YES | **v) REASSURANCE accepted** □ 0= NO, □1 = YES |
| **V19) WILLINGNESS TO RECEIVE HELP**  □ 0= NO, □1 = YES | **V44/15) ABSENCE OF SOCIO-ECONOMIC DISTRESS** (Absence of social risk as housing isolation, disability or illness extra COVID) □ 0= NO, □1 = YES |
| **SOCIAL SUPPORT RESOURCES ____ / 4** | |
| **v18/v39) FAMILIAR SUPPORT** □ 0= NO, □1 = YES | **V40)** **FRIENDS’ SUPPORT** □ 0= NO, □1 = YES |
| **v41) RELIGIOUS SUPPORT** □ 0= NO, □1 = YES | **V42) PROFESSIONAL SUPPORT** □ 0= NO, □1 = YES |

**2.C) INTERVENTION TECHNIQUES**

| **V8) INTERVENTION TECHNIQUES used** | **1**  **session** | **2**  **session** | **3**  **session** | **4**  **session** |
| --- | --- | --- | --- | --- |
| Containment (stabilization) | □ | □ | □ | □ |
| Normalization (stabilization) | □ | □ | □ | □ |
| Psychoeducation | □ | □ | □ | □ |
| Coping strategies and stress reduction (defusing, debriefing, EMDR protocols) | □ | □ | □ | □ |
| Counseling | □ | □ | □ | □ |
| Communication of the bereavement/Structured support in processing bereavement | □ | □ | □ | □ |
| Referring (Referral to another service) | □ | □ | □ | □ |
| Useful COVID-19-related information | □ | □ | □ | □ |
| Network intervention | □ | □ | □ | □ |

**2.D) INDICATIONS FOR ACTION and PRIORITY OF INTERVENTION**

| **V17) INDICATIONS FOR ACTION** | | | |
| --- | --- | --- | --- |
| **ACUTE PHASE** | □ HEALTH INTERVENTION | □ Continue with EPE | □ Department of Mental Health and Pathological Addictions |
|  | □ Other …………… | | |
| **SHORT-LONG-TERM** | □ HEALTH  INTERVENTION | | □ Department of Mental Health and Pathological Addictions |
|  | □ Other …………… | | |

| **V12)** **TRIAGE AND/OR PRIORITY OF INTERVENTION (v20)** | | |
| --- | --- | --- |
| □ **GREEN CODE** = 1  Symptoms are mild and do not require urgent intervention | □ **YELLOW CODE** = 2  Symptoms requiring monitoring | □ **RED CODE =** 3  Severe symptoms require immediate specialized intervention and/or immediate evaluation |

**PART 3: for the last session with EPE team**

**V19) PSYCHO-SOCIAL SUPPORT SCALE (PSSS) – English Version**

For each of the following sentences, mark the answer that best describes how you have felt over the past few days, including today.

|  |  | 0 | 1 | 2 | 3 | 4 |
| --- | --- | --- | --- | --- | --- | --- |
|  |  | **Not at all** | **A little** | **Somewhat** | **A lot** | **Very much** |
| (v45) | 1) I felt helped by others | **□** | **□** | **□** | **□** | **□** |
| (v46) | 2) I felt understood by others | **□** | **□** | **□** | **□** | **□** |
| (v47) | 3) I was able to talk to others | **□** | **□** | **□** | **□** | **□** |
| (v48) | 4) Someone has helped me and/or is helping me solve my daily problems | **□** | **□** | **□** | **□** | **□** |
| **(v49)** | **Satisfaction for the aid by the EPE** |  |  |  |  |  |
|  |  | **Not at all** | **A little** | **Somewhat** | **A lot** | **Very much** |
|  | 5) How helpful do you think our psychological support has been to you? | **□** | **□** | **□** | **□** | **□** |

**PSYCHO-SOCIAL SUPPORT SCALE - VERSIONE ITALIANA***Panzeri A. et al. 2022*

| **Istruzioni**: Legga le seguenti frasi e per ognuna segni la risposta che meglio descrive come si è sentito in questo periodo. Faccia riferimento agli ultimi 15 giorni, compreso oggi, e scelga la sua risposta tra queste. | | | | | | |
| --- | --- | --- | --- | --- | --- | --- |
| # | Item | **Per nulla** | **Poco** | **Abbastanza** | **Molto** | **Moltissimo** |
| 1 | Mi sono sentito aiutato dagli altri | 0 | 1 | 2 | 3 | 4 |
| 2 | Mi sono sentito capito dagli altri | 0 | 1 | 2 | 3 | 4 |
| 3 | Sono riuscito a parlare con gli altri | 0 | 1 | 2 | 3 | 4 |
| 4 | Qualcuno mi ha aiutato a risolvere i miei problemi personali | 0 | 1 | 2 | 3 | 4 |
|  |  |  |  |  |  |  |
|  |  |  |  |  | **Totale:** | **______ /16** |

Nota: Per calcolare il totale, sommare i punteggi degli item.
 Il punteggio massimo è 16.
